# OP44 Artificial Intelligence-Based Mobile Health Technology For Improved Access To Dermatological Care: A Kenyan Case Study

**DOI:** 10.1017/S0266462325101025

**Published:** 2025-12-29

**Authors:** Carme Carrion, Aïna Fuster-Casanovas, Emily Quilter, Mireia Cano, Ruth Nyangacha, Esther Kinyeru, Noemí Robles, Queralt Miró-Catalina, Josep Vidal-Alaball, Antoni Pérez-Navarro, Marta Aymerich, José Antonio Ruiz Postigo

## Abstract

**Introduction:**

Skin-related neglected tropical diseases (skin NTDs) are very prevalent in endemic areas. Resources to manage them are very scarce. The World Health Organization’s Skin NTDs app is designed to help frontline health workers in identifying skin NTDs (n=13) and common skin conditions (n=24). A beta version including artificial intelligence (AI) was developed, and its accuracy and usability was assessed in real life conditions.

**Methods:**

The Skin NTDs app usability and user experience was assessed in frontline healthcare workers (n=38) in Kenya. Participants answered the user Mobile App Rating Scale (uMARS) questionnaire. Focus group discussions (n=4) and semi-structured interviews (n=15) were used to get an in-depth understanding of the user experience. To assess accuracy of the AI algorithm, 40 participants from five counties in Kenya used the app for five months, uploading photographs of the skin lesion (n=605) to an external platform. AI algorithm accuracy was calculated based on the gold standard of consensus diagnosis reached by three independent dermatologists.

**Results:**

The Skin NTDs app received high scores on the uMARS questionnaire (mean app quality 3.82/5 and perceived impact 4.1/5; n=38). Focus group discussions and interview responses aligned with the uMARS findings, reinforcing the positive assessment of the app. It helped to empower professionals, increased their knowledge about skin diseases, and improved their communications skills with patients. The app was time saving and reduced referral of patients to specialists. Some features to be improved were identified. Overall accuracy of the app was found to be 80 percent in diseases where AI has been trained with a higher number of photos of endemic skin conditions.

**Conclusions:**

The Skin NTDs app showed commendable quality and holds potential to be scaled up and implemented at the national level in Kenya and globally. It performs well as a clinical decision support system to help frontline healthcare workers identify potential skin diseases that patients suffer from and to reduce the number of referrals to dermatologists in contexts where there is a lack of specialized professionals.

